# DNA Methylation in *INA*, *NHLH2*, and *THBS4* Is Associated with Metastatic Disease in Renal Cell Carcinoma

**DOI:** 10.3390/cancers14010039

**Published:** 2021-12-22

**Authors:** Olga Katzendorn, Inga Peters, Natalia Dubrowinskaja, Joana M. Moog, Christel Reese, Hossein Tezval, Pouriya Faraj Tabrizi, Jörg Hennenlotter, Marcel Lafos, Markus A. Kuczyk, Jürgen Serth

**Affiliations:** 1 Department of Urology and Urologic Oncology, Hannover Medical School, 30625 Hannover, Germany; katzendorn.olga@mh-hannover.de (O.K.); peters.inga@mh-hannover.de (I.P.); dubrowinskaja.natalia@mh-hannover.de (N.D.); moog.joana@googlemail.com (J.M.M.); Reese.Christel@mh-hannover.de (C.R.); tezval.hossein@mh-hannover.de (H.T.); FarajTabrizi.Pouriya@mh-hannover.de (P.F.T.); kuczyk.markus@mh-hannover.de (M.A.K.); 2 Department of Urology, Eberhard Karls University of Tuebingen, 72076 Tuebingen, Germany; Joerg.Hennenlotter@med.uni-tuebingen.de; 3 Department of Pathology, Hannover Medical School, 30625 Hannover, Germany; Lafos.Marcel@mh-hannover.de

**Keywords:** renal cell carcinoma, metastasis, NHLH2, INA, THBS4, hypermethylation, signature, prognosis

## Abstract

**Simple Summary:**

Early prediction of aggressive cancer using biomarkers is thought to be important for future improvements in the personalized treatment of renal cell carcinoma (RCC). Epigenetic alterations, such as DNA methylation, are promising candidates because they are frequently associated with distant metastasis of tumors and combinations of them offer greater informativity. Here, we describe the indication of the metastatic disease state of renal cell tumors by the methylation of three new candidate genes, *INA, NHLH2*, and *THBS4*. The inclusion of the methylation status of these genes could improve the early detection of metastatic tendency in renal tumors and help identify patients who will benefit from adjuvant treatments.

**Abstract:**

The detection of DNA methylation in primary tumor tissues could be relevant for early stratification of aggressive renal cell carcinomas (RCCs) as a basis for future personalized adjuvant therapy. Methylated TCGA KIRC based candidate CpG loci in *INA, NHLH2*, and *THBS4* that are possibly associated with RCC metastasis were evaluated by pyrosequencing in 154 paired normal adjacent and primary tumor tissues, as well as in 202 metastatic tissues. Statistical analysis was carried out by bivariate logistic regression for group comparisons, log rank survival analysis, and unsupervised and supervised analysis for the classification of tumors. Increased methylation of *INA, NHLH2*, and *THBS4* loci were significantly associated with distant metastasis in primary tumors (*p* < 0.05), tissue-specific hypermethylation in metastatic (*p* = 7.88 × 10^−8^, 5.57 × 10^−10^, 2.06 × 10^−7^) and tumor tissues (*p* = 3.72 × 10^−24^, 3.17 × 10^−13^, 1.58 × 10^−19^), and shortened progression free survival in patients (*p* = 0.03). Combined use of CpG site-specific methylation permits the discrimination of tissues with metastatic disease and reveals a significant contribution of CpG sites in all genes to the statistical classification model. Thus, metastasis in RCC is significantly associated with methylation alterations in *INA, NHLH2*, and *THBS4* loci, providing independent information for the potential early detection of aggressive renal cancers as a rationale for stratifying patients to adjuvant therapies.

## 1. Introduction

Renal cell carcinoma (RCC) is the most common kidney neoplasia and is observed in 5% and 3% of all cancer diagnoses in men and women, respectively [1]. Although the majority of RCCs are diagnosed incidentally in early stages of the tumor on abdominal imaging, up to 25% of patients present with metastases at the time of diagnosis [2]. Though therapeutic approaches have evolved substantially in the last few decades, the 5-year survival of patients with metastatic disease is still poor [3,4]. Clinical predictions and risk stratification for localized and metastatic RCC currently rely on combined clinical, histological, and laboratory parameters summarized in risk score models, such as the stage, size, grade, and necrosis (SSIGN) score or the University of California Integrated Staging System (UISS). In contrast, biomarker stratification for disease prognosis or prediction is still at the beginning of development, and available clinical scores or biomarkers exhibit a lack of validation for non-metastatic localized RCC [5,6,7,8]. Taking into account that even patients with a clinicopathologically defined localized disease may develop aggressive disease, the need for biomarkers able to guide subsequent clinical decisions based on personalized adjuvant treatment strategies, appears to be evident [6,9]. This is further underlined by recent therapeutic advances demonstrating that subsets of localized RCC with a higher recurrence risk (tumor stage T ≥ 2, differentiation ≥ G3) likely benefit from adjuvant therapy with pembrolizumab, a PD-1 checkpoint inhibitor [10]. Moreover, considering that approximately one-third of these patients present with grade 3 and higher adverse events, individualized biomarker-based prediction of response or non-response could reduce non-beneficial treatments [10,11].

Recently, The Cancer Genome Atlas (TCGA) project substantially contributed to a detailed molecular characterization of RCC, providing substantial information about genetic and epigenetic alterations in different histological subtypes of RCC, such as the most common entity described in the Kidney RCC (KIRC) database, clear cell RCC (ccRCC) [12,13]. The KIRC project reported highly individual mutation profiles, indicating that the use of prospective clinical detection of mutations in risk stratification models and personalized therapeutic approaches is basically limited [12]. In contrast, DNA methylation has not only been demonstrated to occur frequently in RCC, but has also been shown to be associated with a number of clinically relevant adverse parameters [14,15]. Therefore, a substantial number of studies, including our work, have revealed specific associations between DNA methylation and unfavorable histopathological characteristics [16,17,18,19,20], metastatic disease [16,17,18,19,21], shorter recurrence-free or cancer-specific survival [15,16,17,19,22,23,24,25] and the predicted response to anti-angiogenic therapy [26,27].

Although a significant number of epigenetic alterations have shown strong associations with clinicopathological parameters, no biomarker or marker panel has been translated into clinical routine. The reasons for this appear to be complex and include circumstances, such as the broad application of retrospective study designs, the limited informativity of single marker studies, and the limited size of patient cohorts, leading to sampling and selection bias and other restrictions [28,29].

To improve the informativity of DNA methylation-based detection and/or prediction of metachronous metastatic disease, we biometrically analyzed the KIRC database and identified internexin neuronal intermediate filament protein alpha (*INA*), nescient helix–loop–helix 2 (*NHLH2*), and thrombospondin 4 (*THBS4*) as candidate genes showing an association between methylation and distant metastasis.

INA is a neuronal cytoskeletal intermediate filament expressed during neuronal development and forms homo- and hetero-polymeric filaments with other neuronal filaments. The physiological function of INA is still unclear [30,31]. In malignant disease, expression of INA at the protein level is associated with better progression free survival (PFS) and longer overall survival (OS) from gliomas and glioblastomas [32,33]. Accordingly, loss of INA expression correlates with adverse histopathological characteristics and worse prognosis in gastroenteropancreatic neuroendocrine neoplasms [34,35]. Moreover, an association of *INA* hypermethylation with worse histopathological characteristics has been described [34]. Interestingly, in gastrointestinal neuroendocrine neoplasms and colorectal cancer, *INA* hypermethylation is associated with a loss of expression, suggesting epigenetic silencing via hypermethylation [34,36].

NHLH2 is a neuronal basic helix–loop–helix (bHLH) transcription factor characterized by a basic DNA binding motif and HLH binding site forming heterodimers or homodimers with other transcription factors [37,38]. NHLH2 has been linked to the regulation of hypothalamic gene expression, body weight control, and hypogonadism in mice [39,40]. In primary neuroblastoma, overexpression of NHLH2 protein is associated with poor patient survival [41].

THBS4 is an extracellular calcium-binding glycoprotein that interacts with cell surfaces or components of the extracellular matrix (ECM) [42]. This protein mediates tissue remodeling and is involved in wound healing and angiogenesis [42,43]. Overexpression of both THBS4 protein and mRNA has been demonstrated in different urological and gastrointestinal solid tumors with increased potency of cellular invasion in vitro [44,45,46]. An association of higher levels of THBS4 protein and worse clinicopathological characteristics has been found in gastric and hepatocellular carcinoma [45,47]. In contrast, other studies have reported lower levels of THBS4 protein and hypermethylation of *THBS4* in colorectal cancer, cutaneous T-cell lymphoma, and bladder cancer with an indication of epigenetic silencing in these tumors [48,49,50].

In the present study, we analyzed DNA methylation of *INA, NHLH2*, and *THBS4* in normal, tumor, and metastatic renal tissue samples and found tissue-specific hypermethylation associated with the metastatic disease state, adverse clinical parameters, and shorter PFS. We also demonstrate that combined information on the methylation signature allows the identification of metastatic disease in unknown tissue samples with good accuracy.

## 2. Materials and Methods

### 2.1. In Silico Analysis to Identify Candidate Loci

Level 3 data from the TCGA KIRC HM450k methylation dataset and statistical software R version 3.6.1 were used to identify metastasis-associated candidate loci in a univariate logistic regression analysis comparing independent tissue sample data between non-metastasized (M0) and metastasized primary tumors (M+) [12,51]. Results were adjusted using the Benjamini–Hochberg correction for multiple statistical testing and ranked by calculating the product of fold-change in group means and the logarithm of reciprocal *p*-values.

### 2.2. Study Design

Gene-wise averaged CpG site-relative methylation values were used to analyze potential associations between gene-related methylation and clinical features in a cross-sectional study. A subset of patients with corresponding data was subjected to analysis of PFS. Moreover, paired tumor-adjacent histopathological normal (adN) and tumor tissues (TU) were compared to detect tumor-specific hypermethylation, and tumor samples with absent distant metastasis status (M0) were compared to an independent tissue cohort of metastatic tissue samples (Mtx) to detect metastasis-specific hypermethylation. Case–control comparisons of primary RCC tissue samples with localized disease and samples with metastatic disease or metastatic tissue samples were used for statistical classification and to determine diagnostic parameters after a random split into equally sized training and test cohorts.

### 2.3. Study Cohort

A total of 189 RCC tumor tissues, 154 paired adN tissues, and 202 metastases from 100 patients with metastatic RCC disease were subjected to methylation analysis. Patient characteristics are summarized in Table 1. The characteristics of the metastatic tissue cohort and the tissue sampling, TNM classification, grading, and tissue treatment were described previously [21,52]. Ethical approval was obtained from the ethical boards of Eberhard Karls University Tübingen and Hanover Medical School (no. 128/2003V and 1213-2011; approved on 14 October 2011). Written informed consent was obtained from all patients. The study was performed in accordance with the Helsinki Declaration.

### 2.4. Nucleic Acid Extraction and DNA Bisulfite Conversion

Histological tumor cell content was estimated in control sections, DNA isolated from frozen sections and punches of formalin-fixed paraffin-embedded tissue samples, and bisulfite conversion of DNA carried out as reported previously [16,23].

### 2.5. DNA Methylation Analysis

**Figure 1:**
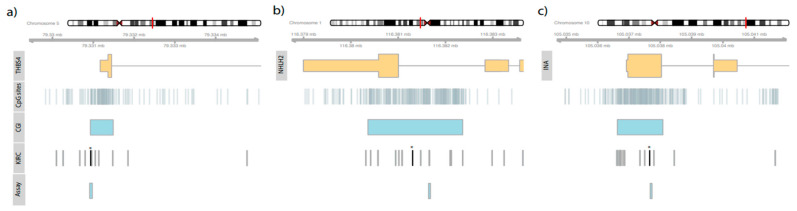
Localization of exons (higher part of orange rectangles) and the genomic regions corresponding to the 5’UTR (*INA* (**c**), *THBS4* (**a**), or 3′UTR (*NHLH2* (**b**)) (lower part of orange rectangles) are shown. CpG sites are annotated for the region (CpG sites), as well as the localization of CpG islands (CGI), positions of CpG sites considered in the KIRC study (KIRC), and the localization of pyrosequencing assays used (Assay). The asterisk (*) tags candidate CpG sites identified in the in silico analysis of the KIRC data.

Pyrosequencing, PCR reactions, and the preparation of pyrosequencing templates were carried out as described previously [16,22]. Pyrosequencing assays were designed by using PyroMark Assay Design 2.0 software (Qiagen, Hilden, Germany) and the hg19 genome assembly as provided by the UCSC table browser. Primer sequences, sequences to analyze, and genomic positions are presented in Table S1. The genomic context of target genes, annotated HM450K CpG sites, candidate loci, and sites covered by the pyrosequencing assay are presented in Figure 1. CpG sites amenable by pyrosequencing analysis and used for subsequent statistical evaluation are summarized in Table 2.

### 2.6. Statistical Analysis

All statistical analyses were performed in R version 3.6.1 software and program libraries as specified below [51]. Statistical tissue group comparisons were carried out using gene-wise aggregated methylation values obtained by calculating the corresponding means for CpG site-specific methylation values. Subgroup evaluations of the association with clinical or pathological parameters were performed in bivariate logistic regression models with age as the covariate and, if necessary, following dichotomization as specified. The time to progression of disease was analyzed by univariate log rank analysis. The optimized cut-off for dichotomization of methylation was approximated using the R package maxstat; relative methylation values for dichotomization were 24% for *INA*, 11% for *NHLH2*, and 25% for *THBS4* [53]. Metastatic tissue samples were compared to independent primary cancer tissues following patient-specific aggregation of measurements obtained for multiple metastases by calculating the mean metastatic tissue methylation value and logistic regression analysis. We applied the two-sided paired t-test to evaluate methylation in tumor and paired adN tissues.

Unsupervised and supervised statistical classification analyses of tissues were carried out by making use of CpG site-specific methylation data including 18 sites annotated to the three candidate gene regions of interest: *INA*, 7 sites; *NHLH2*, 4 sites; and *THBS4*, 7 sites. Missing data for unsupervised clustering analysis were imputed using the mice package for R [54]. Comparisons of the efficiency of various clustering methods, including hierarchical agglomerative, divisive top-down clustering, and partitioning methods were carried out using the ClusterTool library and estimated Jaccard indices applied as a measure of the cluster stability [55]. Consensus clusters of 100 runs are presented as heatmaps using the R-package ComplexHeatmaps [56]. Explorative statistical analyses of the association between cluster class and metastatic disease state were carried out using the R package vcd [57]. For supervised statistical analyses, missing data were treated in a two-step procedure by sequentially removing patient samples with the maximum numbers of missing values and imputing residual absent values using the mice package for R [54]. Random forest classification analysis was carried out following optimization of the random forest model parameters using the R libraries caret and ranger [58,59,60]. The final model diagnostic parameters were determined using the caret package [58]. The importance of variables was calculated and presented using the randomForestExplainer package [61].

## 3. Results

### 3.1. In Silico Identification of Metastasis-Associated Methylated Loci Using KIRC Data

KIRC methylation data for 282 tumor tissues were subjected to univariate logistic regression analysis comparing the subsets of 230 M0 and 52 M+ tumors. Statistical evaluation identified CpG sites cg00824018, cg00065905, and cg00795341, which were annotated to *INA, NHLH2*, and *TBHS4*, respectively, as being among the top 20 ranked candidates. Associated mean fold changes in methylation were 1.68, 1.55, and 2.01 with corresponding *p*-values of 1.30 × 10^−34^, 1.72 × 10^−34^, and 1.90 × 10^−19^, respectively. A literature inquiry presented evidence of a functional or statistical association with tumorigenesis. Following a technical evaluation, whether the loci of the corresponding candidate genes are expected to be accessible to DNA methylation analysis was determined by pyrosequencing. Features of the pyrosequencing assays are summarized in Table S1 and illustrated in Figure 1.

### 3.2. Association of INA, NHLH2, and THBS4 CpG Site Methylation with Adverse Clinicopathological Parameters and Worse PFS

A statistical comparison of tumors with ccRCC and papillary histological classification using bivariate logistic regression including age as a covariate exhibited a negative association for the *INA* loci (*p* = 0.04, odds ratio (OR) = 0.95, 95% confidence interval (CI): 0.91–0.99), whereas *THBS4* loci methylation exclusively demonstrated an association with patient sex in the whole cohort (*p* = 0.04, OR = 1.03, 95% CI: 1.00–1.05) but not the ccRCC subgroup (*p* = 0.09, OR = 1.03).

**Figure 2:**
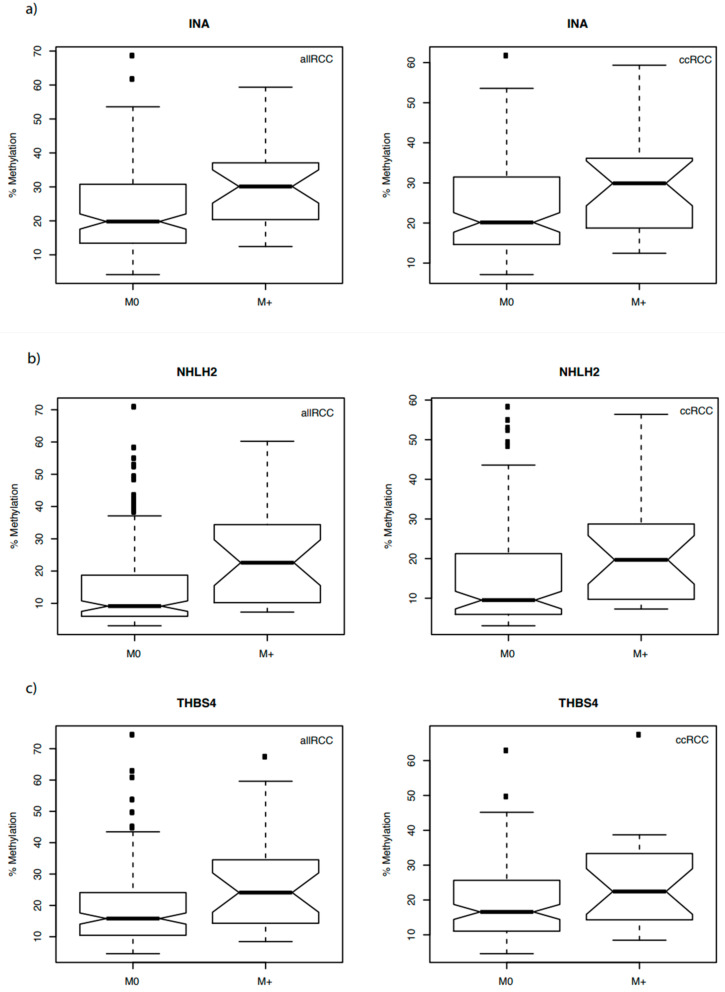
Box plot of *INA* (**a**), *NHLH2* (**b**), and *THBS4* (**c**) methylation in tumors without (M0) and with metastatic disease (M+) for the complete cohort (allRCC) and ccRCC subgroups. Medians are shown with the estimated confidence interval (notches), 25% and 75% quartiles. Whiskers indicate the 99.3% interval (two-sided 1.5-fold interquartile range) and black squares the outliers for the relative methylation distributions. Corresponding *p*-values and odds ratios are presented in Table 3.

**Figure 3:**
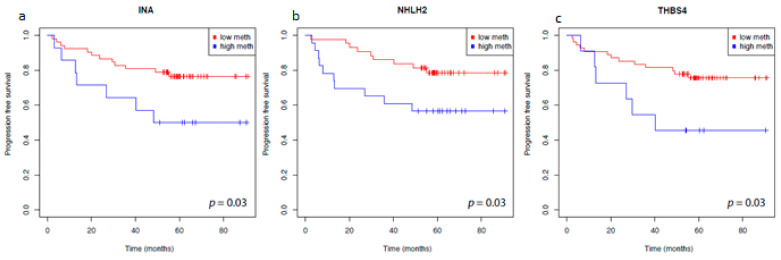
Kaplan–Meier survival analysis of *INA (***a**)*, NHLH2* (**b**) and *THBS4* (**c**) and progression free survival. Blue curve, patients with methylation levels above the gene-specific optimum cut-off. Red curve, patients with methylation levels below the gene-specific optimum cut-off. Optimized cut-off values applied for dichotomization were 24% (*INA*), 11% (*NHLH2*), and 25% (*THBS4*).

Therefore, we analyzed the possible associations between the candidate genes and the state of distant metastasis as the most relevant clinical parameter for both the complete cohort of tumors independent of the histological state of tissues (allRCC group) and the larger subset of clear cell tumors (ccRCC group). Notably, all of the candidate genes showed a significant association between methylation and the state of distant metastases (Figure 2a–c). Moreover, all of the clinical parameters widely used to estimate the aggressiveness of tumors, including tumor stage, status of lymph node metastasis, and grade of differentiation were significantly associated with higher methylation of the *INA, NHLH2*, and *THBS4* candidate loci in both the allRCC and ccRCC groups (Table 3a,b). Survival analysis using available follow-up data for a subset of tumors demonstrated a possible significant association of higher tumor methylation and shortened time to disease progression in univariate log rank analyses for *INA, NHLH2*, and *THBS4* methylation (Figure 3).

### 3.3. INA, NHLH2, and THBS4 Associated Candidate Loci Exhibit Tissue-Specific Hypermethylation in Tumor and Metastatic Tissues

**Figure 4:**
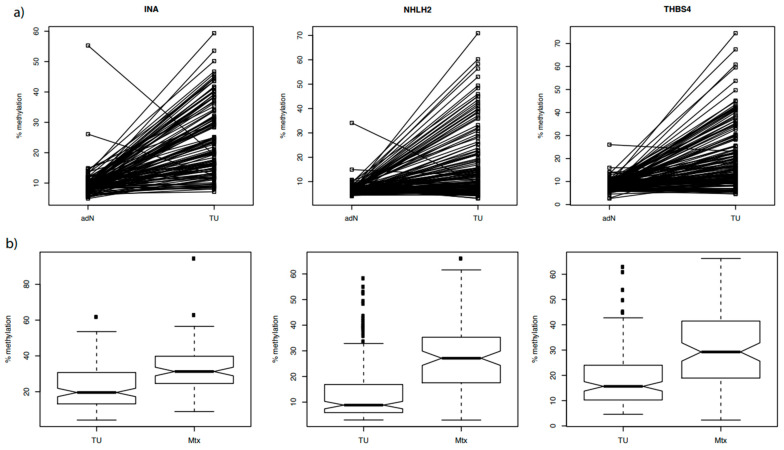
(**a**) Strip chart presentation of the hypermethylation analysis of *INA, NHLH2*, and *THBS4* in paired adjacent normal tissue (adN) and renal tumors (TU) samples. The corresponding statistical analysis revealed significant tumor-specific hypermethylation for all candidate genes (*p* = 3.72 × 10^−24^, 3.17 × 10^−13^, 1.58 × 10^−19^). (**b**) Box plot of *INA, NHLH2*, and *THBS4* methylation in independent tissue sample groups representing localized primary tumor and metastatic tissue samples. All of the candidate genes demonstrated metastatic tissue-specific hypermethylation (*p* = 7.88 × 10^−8^, 5.57 × 10^−10^, 2.06 × 10^−7^ respectively).

To analyze whether the candidate genes have tumor-specific hypermethylation, we measured 120, 141, and 132 corresponding pairs of adN and tumor tissue specimens for methylation of *INA, NHLH2*, and *THBS4* (Figure 4a). Evaluation of INA was limited to the ccRCC tissue pairs due to a possible effect of histology on methylation. In contrast, *NHLH2* and *THBS4* could be analyzed independent from tumor histology. We found significant tumor-specific hypermethylation for all of the candidate genes in the paired tissue comparisons (*p* = 3.72 × 10^−24^, 3.17 × 10^−13^, 1.58 × 10^−19^).

Methylation of the *INA, NHLH2,* and *THBS4* loci was also measured in a subset of 136–142 primary tumor tissues free of lymph node or distant metastasis and a total of 202 cancer metastatic tissues isolated from 100 renal cell cancer patients suffering from metachronous metastatic disease (Figure 4b, Table 4). Relative fold-changes in mean methylation of 1.44, 1.91, and 1.57 was detected for the metastatic tissue groups compared to localized primary tumor tissues, showing uniformly metastasis-specific hypermethylation for each of the candidate genes (*p* = 7.88 × 10^−8^, 5.57 × 10^−10^, 2.06 × 10^−7^). Interestingly, methylation as observed in some of the multiple metastases available for a subset of primary tumors was heterogeneous and reached high methylation values of more than 80% overall. 

### 3.4. Combined CpG Site-Specific DNA Methylation Is Informative of the Metastatic Disease State in Unsupervised and Supervised Statistical Analysis

To investigate whether methylation levels provide information supporting molecular identification of renal tumor tissues exhibiting metastatic disease (i.e., primary tumor tissue with proven M+ state or Mtx), a CpG loci-centric evaluation was carried out by performing both unsupervised and supervised classification of samples.

**Figure 5:**
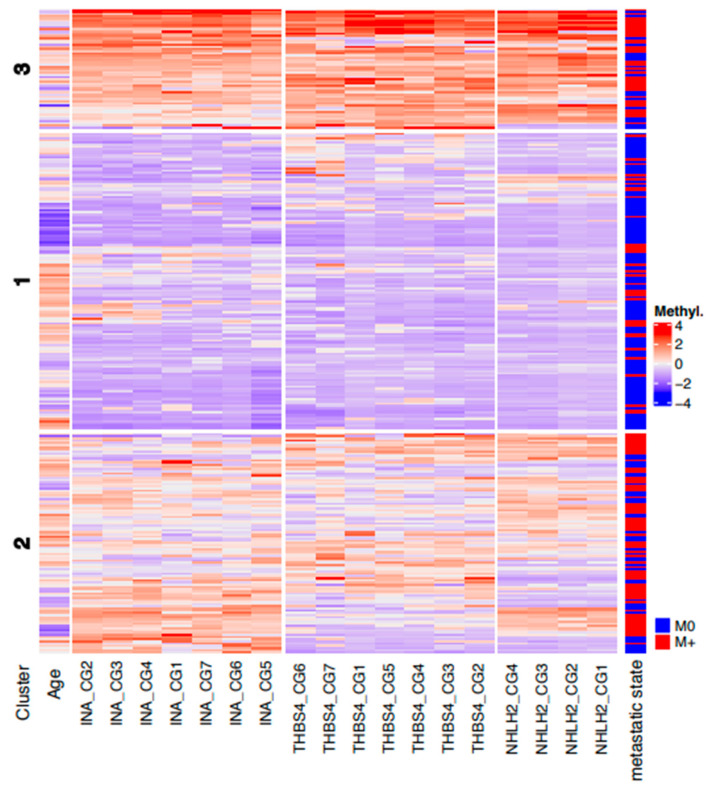
Heat map of unsupervised partitioning of the CpG site-specific methylation as observed in all tumor and metastatic tissue samples. Rows show patient- and sample-specific methylation data following normalization and color coding as indicated (from blue: minimum methylation to red: high methylation). The three patient clusters show most stable consensus clusters obtained by k-means partitioning. Columns present the clustered normalized ages of patients and CpG site-specific methylation data. For comparison sample corresponding metastatic disease state (M0: localized primary tumors, M+: primary metastatic tumors or metastatic tissue samples) are presented.

**Figure 6:**
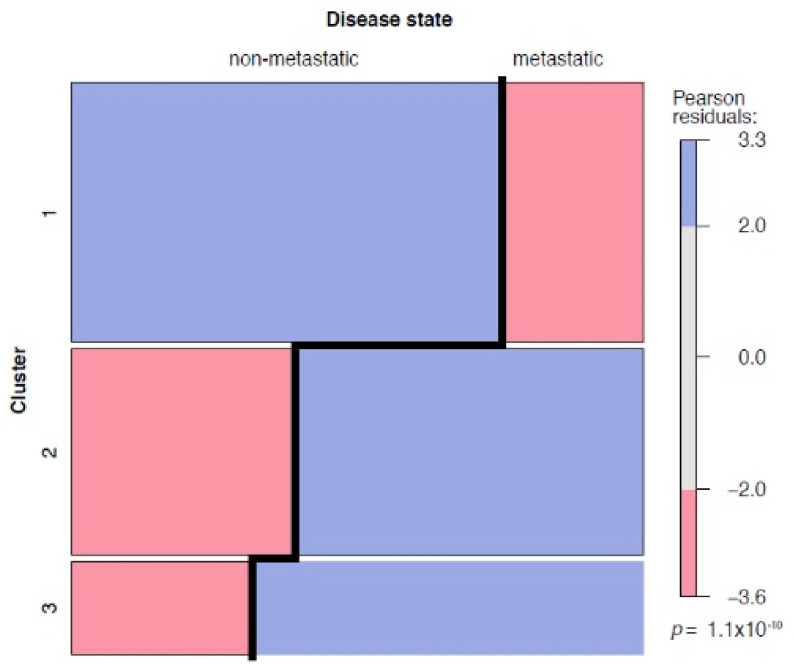
Left and right columns show the associations of clusters with either non-metastatic (left column, primary tumor M0 group) or metastatic disease state (right column, primary tumor M+ group and metastatic tissue Mtx group). Blue boxes indicate over-represented disease states in a cluster (Pearson residuals > 2), whereas red boxes correspond to under-represented disease states (Pearson residuals < −2), providing statistical evidence that clustering is not independent from the metastatic disease state (*p* = 1.1 × 10^−10^).

Unsupervised analysis was carried out following a comparison of various clustering and partition methods aiming to identify the most stable sample clusters, including bootstrapping and cross validation of samples. We found that k-means partitioning with three centers provided the most stable patient clusters, with Jaccard indices of 0.96, 0.88, and 0.81, respectively. A heatmap of the corresponding k-means clustering (Figure 5) shows the three patient consensus clusters, approximately characterized by low (cluster 1), median (cluster 2), and high (cluster 3) average methylation levels. Statistical analysis of the corresponding matching table (Table 5) revealed that patient clusters were not independent from the metastatic disease state of tissues (*p* = 1.1 × 10^−10^, chi-squared test), and the corresponding mosaic plot demonstrated that clusters 2 and 3 included higher fractions of metastatic RCC tissues (Figure 6, blue groups in clusters 2 and 3 for metastatic disease column), whereas cluster 1 contained significantly more M0 tumors than expected by chance (Figure 6, blue group in cluster 1 for non-metastatic disease column). Interestingly, clustering of CpG sites in the heatmap (Figure 5) demonstrated that, in general, the smallest distances were observed for directly neighboring CpG loci and, thus, in large part matched the physical neighborhoods of CpG sites.

Supervised statistical analysis required prior completion of data by imputing 29 of 2140 (1.36%) and 35 of 2160 (1.62%) missing values in the training and test cohorts, eventually providing 107 (58 tumors, 49 metastases) and 108 (61 tumors, 47 metastases) final cases for analysis. Applying the optimized random forest classification model obtained by resampling the training cohorts (25 bootstrapped repetitions, mtry = 2, splitrule = gini) to the unknown test data, a comparison of the real and predicted status of the localized tumor or metastatic tissue in a confusion table revealed 35 true positive, 16 false positive, 45 true negative, and 12 false negative classifications. Therefore, prediction of the independent test cohort demonstrated an accuracy of 0.73 (95% CI: 0.64–0.81, *p* = 4.93 × 10^−6^, one-sided binomial test in which accuracy is greater than the no information rate of 0.52). Classifiers did not demonstrate different proportions of errors for the training and test data (*p* = 0.46, McNemar´s test). Sensitivity and specificity were 0.74 (95% CI: 0.61–0.85) and 0.72 (95% CI: 0.60–0.82), respectively. The positive likelihood ratio (PLR) and negative likelihood ratio (NLR) were 2.67 and 0.35, respectively.

Importance analysis of the variables suggested that 6 of the 7 (86%) *INA*, 2 of the 4 (50%) *NHLH2*, and 2 of the 7 (29%) *THBS4* annotated CpG loci were the most important predictors in the random forest classification model when using the mean decrease in gini and times_a_root parameters as measures (Figure S1).

## 4. Discussion

In the present study, we analyzed whether candidate markers, DNA methylated loci in the *INA, NHLH2*, and *THBS4* genes, show an association with metastasis in RCC and could contribute to predicting the metastatic potential of renal cancer by providing new information on the biomarker signatures of aggressive disease.

In a gene-centric statistical evaluation of *INA, NHLH2,* and *THBS4* methylation data, we found a uniform association of increased methylation with both the metastatic state of primary tumors and the metastatic tissue itself. Therefore, findings from in silico analyses of the TCGA KIRC data were confirmed for all of the candidate genes in independent tissue cohorts and by using a different method of methylation detection. Interestingly, this could also be achieved for the *NHLH2* loci, despite some genomic distance between the pyrosequencing assay and candidate loci location. Neither the KIRC study nor the large part of molecular analysis of RCC include an analysis of metastatic tissue from primary RCC. Thus, to the best of our knowledge, only two studies have reported metastatic tissue-specific alterations in the DNA methylation of *RUNX3* and *TBR1* [21,62]. Although our findings of metastatic hypermethylation of *INA, NHLH2*, and *THBS4* do not allow functional conclusions, it seems that cells carrying these epialterations are preserved, or even expanded, in RCC metastases. Therefore, the corresponding genes are natural candidates for further studies aiming to analyze epithelial–mesenchymal or mesenchymal–epithelial transitions. In addition, the hypothetical functional relevance is supported by our finding of the tumor-specific hypermethylation of all three candidates considering that hypermethylation is frequently followed by epigenetic silencing of tumor suppressor genes and subsequent functional changes in RCC [15]. In line with this, gene silencing by hypermethylation and an association with more invasive tumors was recently reported for *INA* and *THBS4* in other tumor entities [36,48]. Considering our finding of both tumor-specific hypermethylation and an additional increase in the methylation of genes in metastatic tissues, the hypothesis that these epialterations may indicate an increased metastatic potential of cells at an early state of tumor development is supported. Notably, all of our statistical analyses interrogating possible associations of candidate loci methylation with adverse clinicopathological parameters, as well as the survival of patients, point to candidate loci methylation as a characteristic of aggressive cancers.

Unsupervised statistical analyses using CpG site-specific methylation data for the *INA, NHLH2*, and *THBS4* loci showed that tissue samples can be partitioned into three stable clusters that are not independent from the metastatic status of tissues. Nevertheless, detailed comparisons of the assigned clusters and metastatic disease state resulted in a number of tumor misclassifications. This had to be expected considering that a subset of tumors and derived metastases are not affected by *INA, NHLH2*, or *THBS4* hypermethylation, automatically increasing the number of false negative classifications. Moreover, heterogeneous methylation patterns may not give rise to distinct and stable clustering, increasing the chance of misclassification within larger but more stable clusters. In an analogous but reversed manner, some false positive classifications may also be traced back to this effect. However, both phenomena could probably be minimized by expanding the sample cohorts. Interestingly, a considerable number of tumors within higher-methylated clusters 2 and 3 exhibit a homogenously high level of methylation but are classified as non-metastatic disease and appear as apparently false positives. Answering the highly relevant question of whether such tumors may be candidates for metachronous metastasis will, unfortunately, require the setup of a specific tissue cohort and, therefore, could not be addressed in the current study.

Methylation panels combining the CpG methylation of different candidate genes were previously reported to be associated with patient survival, with more robust statistical results and improved performance compared to clinical prognostic models [25,63,64,65]. Moreover, in view of the complex molecular architecture of alterations observed during tumorigenesis and metastasis and the individual variation of tumors, biomarker signatures that are subject to subsequent continuous extension, reevaluation, and reselection, rather than single markers, may be useful for a specific diagnostic task. Our supervised classification analysis of single CpG site methylation information using independent training and test data subsets and random forest analysis revealed that methylation information for a subset of 10 candidate loci from the total of 18 *INA, NHLH2*, and *THBS4* annotated CpG sites already permits the identification of tissue samples with a metastatic disease state in an otherwise unknown tissue cohort with good accuracy. The diagnostic parameters obtained for supervised classification of the test samples are already roughly within the framework of parameters that are characteristic for medical tests [66]. Thus, the inclusion and selection of additional informative candidate loci seems to be a realistic means to achieve the level of accuracy required in order to translate the parameters into widely used diagnostics. The potential value of such measurements has decisively improved with recent findings showing that adjuvant treatment with pembrolizumab is beneficial for RCC patients with high-risk tumors [10]. Thus, future involvement of methylation-based signatures in prognostic models have the potential to improve risk stratification for adjuvant treatments [10,65].

## 5. Conclusions

In conclusion, our analyses demonstrate the informativity of *INA, NHLH2*, and *THBS4* CpG methylation for tissue-based prediction of the metastatic potency of RCC tissues. Thus, these epigenetic markers may be part of a potential tissue biomarker signature for the detection of aggressive disease development in localized RCC.

## Figures and Tables

**Table 1:** Patient’s clinical and histopathologic characteristics.

Clinical and Pathological Characteristics		Total Number of Patients (allRCC), *n* (%)	Subset of Patients with ccRCC, *n* (%)	Subset of Patients with FU for allRCC, *n* (%)	Subset of Patients with FU for ccRCC, *n* (%)
	Total cases	189 (100.0)	151 (100.0)	77 (100.0)	57 (100.0)
Histology	ccRCC	151 (79.9)	151(100.0)	57 (74.0)	57(100.0)
	pap. RCC	25 (13.2)	0 (0.0)	17 (22.1)	0 (0.0)
	chrom. RCC	3 (1.6)	0 (0.0)	2 (2.6)	0 (0.0)
	Mixed histology *	10 (5.3)	0 (0.0)	1 (1.3)	0 (0.0)
Sex	Female	70 (37.0)	59 (39.1)	27 (35.1)	22 (38.6)
	Male	119 (63.0)	92 (60.9)	50 (64.9)	35 (61.4)
Age (years)	Median	65	65	65	64
	(min-max)	(35–91)	(35–90)	(37–91)	(37–90)
Metastasis	M0	156 (82.5)	123 (81.5)	59 (76.6)	41 (71.9)
	M+	30 (15.9)	25 (16.6)	18 (23.4)	16 (28.1)
	na	3 (1.6)	3 (2.0)	0 (0.0)	0 (0.0)
Lymph node status	N0	168 (88.9)	136 (90.1)	70 (90.9)	52 (91.2)
	N+	16 (8.5)	10 (6.6)	7 (9.1)	5 (8.8)
	na	5 (2.6)	5 (3.3)	0 (0.0)	0 (0.0)
Tumor stage	pT1	11 (5.8)	8 (5.3)	8 (10.4)	5 (8.8)
	pT1a	62 (32.8)	48 (31.8)	24 (31.2)	15 (26.3)
	pT1b	46 (24.3)	38 (25.2)	13 (16.9)	9 (15.8)
	pT2	9 (4.8)	7 (4.6)	6 (7.8)	5 (8.8)
	pT3	5 (2.6)	2 (1.3)	2 (2.6)	1 (1.8)
	pT3a	17 (9.0)	14 (9.3)	4 (5.2)	4 (7.0)
	pT3b	32 (16.9)	30 (19.9)	18 (23.4)	17 (29.8)
	pT3c	4 (2.1)	3 (2.0)	2 (2.6)	1 (1.8)
	pT4	1 (0.5)	0 (0.0)	0 (0.0)	0 (0.0)
	na	2 (1.1)	1 (0.7)	0 (0.0)	0 (0.0)
Differentiation	G1	36 (19.0)	32 (21.2)	14 (18.2)	12 (21.1)
	G1–2	20 (10.6)	13 (8.6)	10 (13.0)	4 (7.0)
	G2	104 (55.0)	82 (54.3)	42 (54.5)	30 (52.6)
	G2–3	10 (5.3)	6 (4.0)	5 (6.5)	5 (8.8)
	G3	18 (9.5)	17 (11.3)	6 (7.8)	6 (10.5)
	na	1 (0.5)	1 (0.7)	0 (0.0)	0 (0.0)

Abbreviations: ccRCC clear cell renal cell carcinoma (RCC); pap. RCC papillary RCC; chrom. RCC chromophobe RCC; FU follow-up; na not available; * mixed histology defined as fractions of different histologic subtypes of RCC (ccRCC + pap. RCC, ccRCC + chrom. RCC).

**Table 2:** Genomic position of CpG sites amenable by pyrosequencing.

Gene	Chromosome	Genomic Position Start	Genomic Position End
*INA*	10	105037691, ~700, ~703, ~706, ~713, ~715, ~719, ~728	105037692, ~701, ~704, ~707, ~714, ~716,~720, ~729
*NHLH2*	1	116381644, ~46, ~48, ~52, ~64, ~67, ~79	116381645, ~47, ~49, ~53, ~65, ~68, ~80
*THBS4*	5	79330930, ~44, ~56, ~58, ~60, ~63, ~69	79330931, ~45, ~57, ~59, ~61, ~64, ~70

**Table 3:** Bivariate logistic regression analysis of association of *INA, NHLH2,* and *THBS4* methylation and clinicopathological characteristics.

**(a) Complete Cohort (allRCC)**						
**Methylation**	***INA***		***nhlh2***		***THBS4***	
	**OR (95% CI)**	***p*** **-value**	**OR (95% CI)**	***p*** **-value**	**OR (95% CI)**	***p*** **-value**
Metastasis (M0 vs. M1)	1.05 (1.02–1.09)	0.002	1.05 (1.02–1.07)	<0.001	1,04 (1.01–1.07)	0.008
Lymph node status (N0 vs. N1)	1.04 (1.00–1.09)	<0.001	1.05 (1.02–1.09)	<0.001	1.04 (1.01–1.07)	0.021
Tumor stage (low vs. high T *)	1.07 (1.04–1.10)	<0.001	1.06 (1.04–1.09)	<0.001	1.05 (1.03–1.08)	<0.001
Differentiation (low vs. high G **)	1.08 (1.04–1.12)	<0.001	1.06 (1.03–1.09)	<0.001	1.05 (1.02–1.08)	<0.001
**(b) ccRCC Subgroup**						
**Methylation**	***INA***		***NHLH2***		***THBS4***	
	**OR (95% CI)**	***p*** **-value**	**OR (95% CI)**	***p*** **-value**	**OR (95% CI)**	***p*** **-value**
Metastasis (M0 vs. M1)	1.05 (1.01–1.09)	0.019	1.03 (1.00–1.07)	0.026	1.04 (1.00–1.08)	0.048
Lymph node status (N0 vs. N1)	na ***	na ***	na ***	na ***	na ***	na ***
Tumor stage (low vs. high T *)	1.07 (1.03–1.10)	<0.001	1.05 (1.02–1.08)	<0.001	1.05 (1.02–1.09)	0.001
Differentiation (low vs. high G **)	1.07 (1.03–1.12)	<0.001	1.05 (1.02–1.08)	<0.001	1.04 (1.01–1.08)	0.021

Note that statistical results of covariate age are not shown as statistical significance was either not reached (ccRCC) group or low ORs of 0.95–0.96 with *p*-values from *p* = 0.02–0.05 were obtained indicating weak model effects. Abbreviations: vs. versus; na not available; OR odds ratio; 95% CI 95% confidence interval * Low defined as T1 and T2; high defined as T3 and T4; ** Low defined as G1, G2; high defined as ≥G3; *** Due to small group sizes no statistics available.

**Table 4:** Statistical association of *INA, NHLH2,* and *THBS4* methylation and metastatic tissue in logistic regression analyses.

Methylation	Gene	Mean Methylation (%)		OR (95% CI)	adjOR	*p*-Value
		Tu	Mtx			
TUvs. Mtx	*INA*	22.8	32.8	1.07 (1.05–1.1)	1.98	7.88 × 10^−8^
	*NHLH2*	14.4	27.4	1.07 (1.05–1.1)	2.47	5.57 × 10^−10^
	*THBS4*	19.4	30.4	1.06 (1.04–1.08)	1.85	2.06 × 10^−7^

Abbreviations: adjOR adjusted odds ratio for difference of group means, OR odds ratio, 95% CI 95% Confidence interval, TU tumor, Mtx metastatic tissue.

**Table 5:** Matching of unsupervised clustering and metastatic disease state.

Cluster	**Patients Without Metastasis (M0)**	**Patients with Metastatic Disease State (M+, Mtx)**
Cluster 1	103	33
Cluster 2	42	66
Cluster 3	15	33

## Data Availability

The anonymized datasets used and/or analyzed during the current study are available from the corresponding author upon reasonable request. Due to the General Data Protection Regulation (Art.5 DSGVO), we are not allowed to share sensitive data within an open data-sharing platform.

## Supplementary Materials

The following are available online at www.mdpi.com/article/10.3390/cancers14010039/s1: Table S1: Primer sequences, sequence to analyze and genomic position of the analyzed region for pyrosequencing, Figure S1: Variable of importance analysis of the random forest classification of tissue samples.
