# Supplementary material for: DNA Methylation in INA, NHLH2, and THBS4 Is Associated with Metastatic Disease in Renal Cell Carcinoma
Source: Cancers (Basel). 2021 Dec 22;14(1):39. doi: 10.3390/cancers14010039 (PMC8750163; doi:10.3390/cancers14010039)
Supplement: Supplementary file 1 [file cancers-14-00039-s001.zip › cancers-1484960-supplementary.pdf]

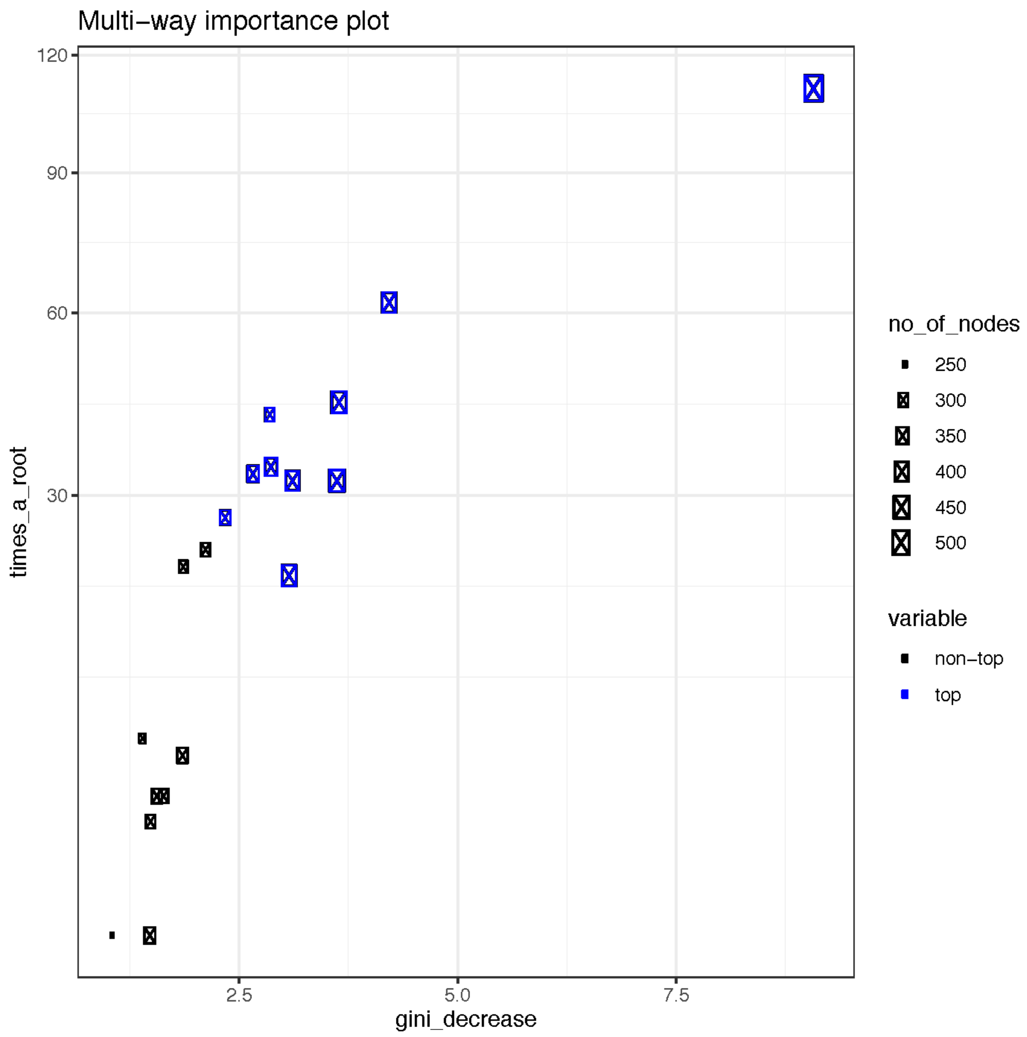

Figure S1. Variable of importance analysis of the random forest classification of tissue samples.

Supplemental Table S1. **Primer Sequences, Sequence to analyse and genomic position of analysed region by pyrosequencing**

Gene	Primer Sequences			Chromosome	Genomic position		Sequence to analyze	Coordinates in UCSC table browser
	Sequencing	Forward	Reverse		Genomic Start	Genomic End		
INA	AAACCCAAATCAAATCTAATTTAAC	AGGAGGTAGTAGAGTTGTTGGTTA	TCTCCCTCAAAACCCAAATCAAATCTAATT	10	105037678	105037737	CACAATCACRTCCACCTCRACCRCRACCTACRACRACRCCTACAACRTAACCAACAACTC	chr10:105037678-105037737
NHLH2	GGGAAGGTTTTTTGGAT	GTTGGGGGAGGGAAGGTT	AACTCCCCCACCTTTATTTAATCATTTCTT	1	116381641	116381682	TTTTYGYGTTTTTTYGAAATYGTAAAGAAATGATTAAATAAA	chr1:116381641-116381682
THBS4	GGGGTGGGTGTAGATA	GAGGATGGGAAGGAGTTTG	TCTCCCCTATAATCTCTACTCAAC	5	79330905	79330970	YGGTTTYGAYGYGYGAGGGTAGGGGYGAAGATGGGTAGTYGGGGTTGGAGAGAAAGGGGTGTTGAG	chr5:79330905-79330970
